# The missing metric: quantifying contributions of reviewers

**DOI:** 10.1098/rsos.140540

**Published:** 2015-02-11

**Authors:** Maurício Cantor, Shane Gero

**Affiliations:** 1 Department of Biology, Dalhousie University, Halifax, Nova Scotia, Canada B3H4J1; 2 Department of BioScience, Aarhus University, Aarhus 8000, Denmark

**Keywords:** peer-review, publication practices, science policy, index, science metrics, research assessment

## Abstract

The number of contributing reviewers often outnumbers the authors of publications. This has led to apathy towards reviewing and the conclusion that the peer-review system is broken. Given the trade-offs between submitting and reviewing manuscripts, reviewers and authors naturally want visibility for their efforts. While study after study has called for revolutionizing publication practices, the current paradigm does not recognize reviewers' time and expertise. We propose the *R-index* as a simple way to quantify scientists' contributions as reviewers. We modelled its performance using simulations based on real data to show that early–mid career scientists, who complete high-quality reviews of longer manuscripts within their field, can perform as well as leading scientists reviewing only for high-impact journals. By giving citeable academic recognition for reviewing, *R*-index will encourage more participation with better reviews, regardless of the career stage. Moreover, the *R*-index will allow editors to exploit scores to manage and improve their review team, and for journals to promote high average scores as signals of a practical and efficient service to authors. Peer-review is a pervasive necessity across disciplines and the simple utility of this missing metric will credit a valuable aspect of academic productivity without having to revolutionize the current peer-review system.

## 2. Introduction

The number of contributing reviewers often outnumbers the authors of publications in several fields. A given manuscript may be reviewed by an average of five to 10 readers before acceptance; often more than once by the same reviewer, and probably by editors from several journals [1]. This has led to apathy among academics towards reviewing and the conclusion by many that the current system of peer-review is broken. Academics have read similar sentences across career generations [2]. Yet, little has changed in contemporary journal culture despite the outcry.

Today, with the rise of online databases, search engines and tracking beyond typical academic institutions, we have the ability to design a measure of scientific contributions through reviewing. While the need to enfranchise reviewers and for quantitative indicators of peer-review have been called for [3–6], none has been operationalized. This is probably because they involve complicated systems of payment or non-financial incentives (e.g. [7–9]), or penalize reviewers' poor performance instead of rewarding their efforts (e.g. [10]).

Here, we propose the *R*-index as a simple way to quantify a scientist's efforts as a reviewer. By its design, it encourages strong reviews for leading journals within one's field and allows editors to manage and measure the reviewers they use. Ultimately, the *R*-index will encourage more and better participation in the peer-review system by researchers of all career stages, credit them proportionally to their efforts and promote transparency in the scientific community.

## 3. Material and methods

### 3.1 The metric

We built on existing impact metrics to propose the *R*-index to quantify referees' contributions to the scientific community:

$$R_{i}=\sum_{j=1}^{J}\sum_{k_{j}=1}^{n_{j}}\left[\sqrt{{\text{IF}}_{j}}\cdot \left(\frac{w_{k_{j}}}{10^{4}}\right)\cdot s_{k_{j}}\right].$$

Each journal, *j*, will disclose their annual list of reviewers, *i*, and the number of papers they reviewed, *n*_*j*_. For each *k*th paper in a given journal *j*, the total number of words, *w*_*kj*_, is multiplied by the square root of the journal's impact factor, IF_*j*_. This product is weighted by the editor's feedback on individual revisions, which is given by a score of excellence *s*_*kj*_ ranging from 0 (poor quality) to 1 (exceptionally good quality).

There is no single measure for time invested in a review; the word count of the submitted manuscript, given by *w*_*kj*_, is an attribute of all manuscripts and is herein considered an intuitive proxy for time spent during each revision. To account for inherent variation in manuscript length of different disciplines, as well as the individual ability to review longer or more methodological texts, we rescaled the word count by 10^−4^. The impact factor IF_*j*_ of the journal to which the manuscript was submitted is herein considered a proxy for the impact of the prospective paper (a truth of our current publication culture), as well as the reviewer's prestige and standing in the field (given that highly recognized scientists are more likely to be invited to review for top-journals). Furthermore, to adjust for disparity across disciplines and career stages, we square rooted the IF values. Finally, editors can promote better reviews and avoid the system to be gamed by ranking each revision with *s*_*kj*_.

We suggest the *s*-score to be standardized across journals by taking the mean value of four qualities of the review, all which range between 0 and 1: punctuality (within or beyond the deadline set by the editor?), utility to authors (are there constructive and specific comments for improving the work?), utility to editors (does the review address all facets of the manuscript—methodological and writing details, adherence to journal format—and is reported clearly and concisely?), and impact (to what degree did the review contribute to the decision made on the manuscript by the editor?). Punctuality can be determined quantitatively by subtracting the proportion of days late from the amount of time given (e.g. if the reviewer is given 30 days and returns it 3 days late, the punctuality score would be 0.9, if it was 15 days late it would be 0.5). The remaining three qualities can be scored on a 5 point scale (e.g. 0: unusable, 0.25: not useful, 0.5: adequate, 0.75: useful, 1.0: very useful). Multi-step Likert-type scales [11] are often used by journals to get reviewers to subjectively quantify the impact, overall quality, broadness of appeal of submitted manuscripts; here, we propose editors use them to quantify similar traits of the reviews themselves. Despite the potential subjectivity of the *s*-score, the scientific community already implicitly trusts the editors' judgement given that it is already their responsibility to evaluate the contribution reviewers to the final paper. Our metric allows for further transparency in this regard.

### 3.2 Metric performance

We modelled the *R*-index performance using simulated data based on real distributions of the index parameters taken from Journal of Citation Reports and from donated anonymized reviewer information (electronic supplementary material, S1 and figure S1). The index formulation aimed to reward reviewers proportionally the contributions to the peer-review system, but taking into account other aspects of it such as standing in the field, time and effort invested. We tested the performance of the index by: (i) considering reduced and alternative versions (SM3), (ii) simulating populations of reviewers with fixed and varying number of reviews *per capita* (SM4), and (iii) simulating reviews performed for journals of varying impact factors (SM4).

We also compared *R*-index outputs across stage careers (SM2) with a large simulated population of 50 000 researchers reviewing 2 875 000 manuscripts: (i) early-career researchers (PhD candidates and post-docs) reviewed many (high *n*) moderately long manuscripts (mid-high *w*) submitted to mid-low rank journals (low IF); (ii) mid-career researchers (more than 10 years after PhD, with permanent positions) reviewed several (moderate *n*) manuscripts of all lengths (normally distributed *w*) submitted to all types of journals (empirically distributed IF); and lead researchers (i.e. professors and related high positions). As the latter is usually invited to review for a broader range of journals and can be more selective, we subdivided them into two strategies: (iii) opportunist leaders, reviewing few (low *n*) and relatively short (moderate-low *w*) manuscripts only for top journals (high IF) and (iv) specialist leaders, reviewing only manuscripts within their area of expertise (low *n*), of all lengths (normally distributed *w*) and submitted to all types of journals (empirically distributed IF). These two strategies aimed to portray the extremes of a range of reviewing habits, between which mixed strategies are expected (electronic supplementary material, figure S2). Number of reviewers and the proportion of accomplished reviews in each of the four categories were based on a recent online survey [12] (see also the electronic supplementary material, S2), which suggested that 54% of reviews are performed by early-career; 32% by mid-career and 14% by lead researchers, while the researcher population is probably composed by 38% of early-career researchers, 39% mid-careers and 23% leaders (Dr J. M. Wicherts 2014, personal communication, editor *PLoS ONE*, corroborates this pattern in an invited review system).

The empirical data on review quality suggest reviewers usually deliver reviews of good quality (empirical *s*-score was *β* distributed; electronic supplementary material, figure S1). To further explore the impact of the editor's feedback (and so the need for providing a good review), we also simulated *R*-index outputs for the different stage careers adjusting the *s*-scores with a stratified sampling of the empirical *s*-score distribution (electronic supplementary material, figure S1). In this scenario, early-career researchers delivered high-quality reviews (i.e. mainly high *s*-scores, sampled from the third and fourth quantiles of the empirical distribution), mid-career and specialist lead researchers delivering good reviews (mid *s*-scores, sampled from the second and third quantiles), and opportunist lead researchers delivering poor reviews (low *s*-scores, sampled from the first and second quantiles of the empirical distribution). Although there are no empirical evidences that reviewers deliver poor reviews, such habit of providing many quick, poor reviews could arise with the implementation of the *R*-index as an attempt to game the system. We aimed to evaluate how this strategy would perform and highlight the importance of providing high-quality reviews. Full details on all simulations are available in the electronic supplementary material.

## 4. Results

Our simulations showed that *R*-index rewards reviewers proportionally to the quantity of reviews, pondered by the reviewer's expertise and time and effort invested in the review (electronic supplementary material, figures S3 and S5). Reduced versions of the index changed its absolute scale but amplified disparities among reviewers of different career stages (electronic supplementary material, figure S4), suggesting that the proposed version was more appropriate to capture and balance out the essentials of the contributions through peer-reviewing.

**Figure 1.**
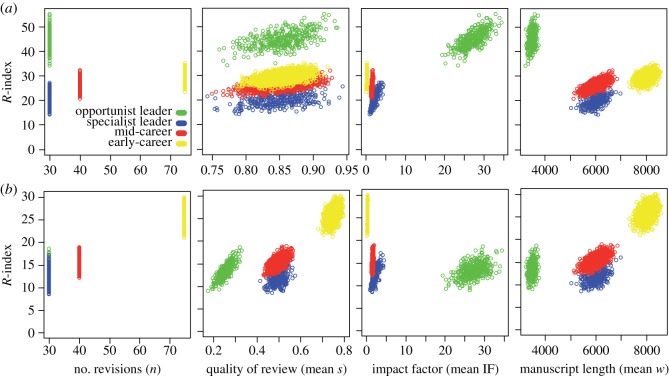
Characteristics of reviews by researchers in different career stages and the importance of review quality. Scatter plots depict simulated *R*-index (*y*-axis) versus mean parameter (*x*-axis) for each reviewer *i* (coloured circles). (*a*) Simulations based on empirical data, which suggest reviewers tend to perform reviews of good quality (empirical *s* *β* distributed; electronic supplementary material, figure S1). (*b*) Simulations with readjusted *s* to emphasize the importance of performing a good review (second column), with early-career researchers delivering high-quality reviews, mid-career and specialist lead researchers delivering good and opportunist lead researchers poor reviews.

**Figure 2.**
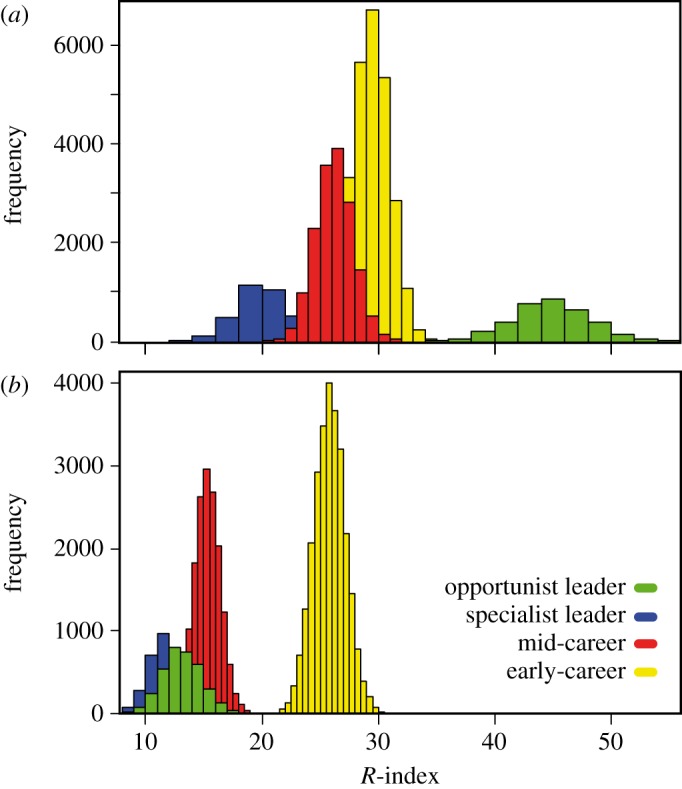
The impact of high-quality reviews on the *R*-index output across career stages. Histograms present the output *R*-index distributions for simulations of figure 1. In (*a*), it is assumed that opportunist leaders, selective leaders, mid-career and early-career researchers deliver good-quality reviews; in (*b*), early-career researchers outperformed other career stages by submitting higher quality reviews.

Assuming that in general, reviewers tend to prepare reviews of good quality (empirical *s*-score distribution, electronic supplementary material, figure S1), our simulations predicted that early-career researchers would have a comparable *R*-index with mid-career and specialist lead researchers (figure 1*a*), mainly owing to the tendency of reviewing more and longer manuscripts in mid-tier, field-leading journals (included in the model based on [12]). In the same scenario, opportunist leaders who review only for high-impact journals would outperform the other strategies (figure 2*a*). However, reviewing for top journals is inherently a rare opportunity (empirical IF distribution; electronic supplementary material, figure S1). Therefore, when we adjusted for the editor's feedback on individual reviews (*s*-score), the *R*-index of early-career researchers who delivered high-quality reviews exceed mid-career and specialist lead researchers delivering good reviews and, most notably, opportunist lead researchers providing poor, quick reviews to the few top journals (figures 1*b* and 2*b*).

Overall, our findings suggested that *R*-index can encourage and give credit to hard-working reviewers who accept and complete quality reviews for field-leading journals, whose manuscripts are typically longer in length, over trying to climb the review pyramid and only reviewing a few times for top journals (figures 1*b* and 2*b*). As the editors' feedback impacts the overall index by rewarding high-quality reviews, the *R*-index is difficult to game. As a result, reviewers must complete five times as many poor reviews to achieve *R*-indices comparable to those submitting quality reviews (electronic supplementary material, figure S6). Similarly, the impact factor of the journal controls for attempts to boost one's *R*-index by completing many poor reviews for predatory, or less reputable, online journals ([13–15]; see electronic supplementary material, figure S7).

## 5. Discussion

The metric outlined here quantifies contributions of academics through review, ultimately making the review process more transparent. We suggest that such a simple method can aid the peer-review system by providing academic recognition to researchers, a tool for editors to manage their review team, and an additional quality standard for journals.

### 5.1 Benefits to reviewers

Given the trade-offs between submitting and reviewing manuscripts, reviewers naturally want visibility for the efforts, just as authors do. *R*-index places emphasis on the importance of review as part of any scientist's productivity. Unlike other proposed grading schemes [3,16,17], *R*-index not only recompenses reviewers proportionally to the quantity but also encompasses their time and effort invested and their standing in the field. Not all journals are equal, but not all reviewers are equal either. In our experience, and we assume that of many, it is often the mid-tier, field-leading journals that produce the most constructive reviews for the authors. Reviewers, and often the editors as well, are typically within the authors' field, have knowledge of the system and the literature and are able to accurately interpret and predict the impact of the work. High *R*-value scientists are our community's unheralded pillars and the *R*-index will provide academic recognition for their contributions.

The current academic employment prospects for early-career scientists are daunting. The pressure to deliver high-impact publications on a repeated basis is driving a culture of metric-based assessment and speculative submissions to leading journals [18]. A recent poll by *Nature* suggests that metrics are perceived as being overweighed in decisions relating to Human Resources; while administration assures that metrics are not important [19]. The truth appears to be that the metrics are inevitably used as a short hand to divide the herd of potential candidates down to a manageable size in which more qualitative, and probably more accurate, measures of assessment can be used such as their letters of recommendation and published work. Currently, the *H*-index [20] is the dominant metric to quantify an individual's scientific publication output. Relating a scientist's output as an author (*H*-index) and a reviewer (*R*-index) would allow for a more holistic view on his or her contributions [21], particularly given that these contributions differ greatly between scientists and most do not do their fair share of reviewing [22]. Early-career scientists, appear to produce significantly more reviews than mid-career or leading professors ([12] and Dr J. M. Wicherts 2014, personal communication, editor at *PLoS ONE*). If there is an implicit expectation for early-career scientist to contribute the bulk of the review load thus credit must be given to those which choose to do so. Our proposed metric makes those choices transparent and easy to evaluate.

### 5.2 Benefits to the journals

The *R*-index is also designed to aid editors and benefit journals. First, it establishes a requirement for scoring reviews—if not already implemented as part of journal policy—with which editors can monitor the reviewers they choose to use. We propose a simple system based on commonly used multi-step Likert-style scales to standardize this measure across journals and disciplines. We believe that all editors strive to have the most qualified and capable reviewers, and *R*-index outputs will allow them a means to do so efficiently.

Second, journals will have two metrics by which to be judged: IF and *R*-index. With concerns in regards to the overuse of IF as a measure of scientific quality [23–26], the journal's mean *R*-index would provide a valuable evaluation of the journals review quality. In the digital age when all publications are almost equally accessible through search engines, there is an increasing disconnection between the IF of the journal a paper is published in and the number of citations that paper eventually receives [26]. As a result, IF is an increasingly less accurate way of evaluating journals, papers and researchers. If reviews are truly improving the quality of publications, then journals would strive to retain high *R*-scored reviewers as an offer to potential submitting authors. It would shift the focus of what the journal is actually offering, from potential, implied prestige to practical aid in the publication process. If the relationship between impact factor and citation continues to decouple and weaken, then authors are likely to seek out journals which provide an efficient and constructive review service prior to publication.

### 5.3 Benefits to the scientific community

Inherently, the *R*-index must increase transparency by encouraging journals to make basic data on reviewers available. Given the mix of support and condemnation for a completely open peer-review system [27–29], this metric is easy to compute and implement within the current pre-publication anonymous system. It is not necessary for a specific connection between reviewers and papers to be made, but simply the disclosure of the number, the quality score of reviews by each scientist and the word length of manuscripts. Pooling of this data within publishers is straightforward and has already begun, and can be used to calculate *R*-index across publishers or even done automatically by leading online databases (e.g. Google Scholar, Web of Knowledge and Scopus). Making such information available allows the scientific community to address the omission of a fundamental contribution to the scientific community, while feeding its growing interest in measuring productivity and reputation in academia.

### 5.4 The ideal of an ‘ideal’ metric

An ideal metric for scientific productivity should be: (i) intuitive, (ii) comparable across fields, (iii) a fair comparator across career stages, and (iv) contain parameters that most of the scientific community support. Take for instance, the nearly ubiquitous *H*-index [20]. It is very intuitive and simple to interpret—an *H* of 20 means one has published 20 papers which have each been cited 20 times—which in many ways is what has led to its pervasiveness. However, the *H*-index fails in several of these criteria: it is not readily comparable across fields with different citing cultures [20,30] nor is it a fair comparator across career stages [30,31].

By contrast, our goal was to operationalize an index that is simple to calculate and yet contains sufficient parameters to capture the reality of an individual's time and efforts as a reviewer. By doing so, the *R*-index gains in broader integrity in quantifying what it is intended to, but it may seem less intuitive than other metrics. An *R*-index of 20 means a solid contribution to the review system, which can be achieved through different ways; to cite a few: (i) 20 excellent reviews of long manuscripts to low rank journals (*n*=20, *s*=1, IF=1, *w*=10 000); (ii) 40 excellent reviews of short manuscripts to low rank journals (*n*=40, *s*=1, IF=1, *w*=5000), (iii) 40 good reviews of short manuscripts to mid rank journals (*n*=40, *s*=0.5, IF=4, *w*=5000); or (iv) 20 very good reviews of short manuscripts to top journals (*n*=20, *s*=0.8, IF=25, *w*=2500). Therefore, the peer-review system is equally benefited from different individual contributions, which makes the *R*-index more diversely applicable and egalitarian as it levels off different reviewers' styles, career stages and disciplines.

As for the final criteria of community acceptance, while one could foreseeably find alternative parameters for our proxies—such as using Source Normalized Impact per Publication [32] instead of IF as a measure of journal calibre and prospective impact of the manuscript or a more complex proxy for time spent per review than manuscript word count—most would only change the absolute value of the *R*-index not the behaviour of the metric itself; and would only lead to a far complex metric. Formulated in the present way, we believe the *R*-index will incentive participation of researchers of all career stages in the peer-review system. More importantly, the *R*-index intends to encourage not only more, but also better, reviews of manuscripts. The *H*-index, and IF before it, created the substrate for discussion and change, arguably negative or positive, in our community in relation to researcher assessment. We believe that the *R*-index quantifying reviewers, and the discussion which follows, will do the same for our peer-review system.

## 6. Conclusion

The current peer-review paradigm does not give reviewers either compensation or citeable academic recognition for their time and expertise. Our proposal converts such hitherto unrecognized ‘obligation’ [33] into a public scale of the contribution of reviewers to the progress of science. Ultimately, implementing the *R*-index will give reviewers citeable academic recognition for their time and expertise, permit hiring and tenure committees to assess their faculty based on a wider truth of academic productivity, and increase transparency within the scientific community. If peer-review is a valuable and under-rewarded endeavour as has been much extolled; then this missing metric allows us to equitably credit those who undertake it.

## Supplementary Material

We are uploading 2 supplementary files, a single pdf document with four sections (SM1: Empirical and simulated data, SM2:. Reviewers' strategies and career stages, SM3: The R-index formulation, SM3: R-index performance) and the binary files for an R package: Cantor_Gero_ESM.pdf

## Supplementary Material

Supplementary methods Rindex_0.1.zip: R package Rindex

## Data accessibility

We provide all the analytical material of this manuscript—the index, the data and scripts for the simulations—in the R package *Rindex*. The binary package has been uploaded as part of the electronic supplementary material and is also available and open-sourced at the repository: https://bitbucket.org/maucantor/rindex. The empirical data are available in the folder */inst/data*; to access the simulated data, please install the package in R and run the simulation scripts.
