# Supplementary material for: The missing metric: quantifying contributions of reviewers
Source: R Soc Open Sci. 2015 Feb 11;2(2):140540. doi: 10.1098/rsos.140540 (PMC4448813; doi:10.1098/rsos.140540)
Supplement: We are uploading 2 supplementary files, a single pdf document with four sections (SM1: Empirical and simulated data, SM2:. Reviewers' strategies and career stages, SM3: The R-index formulation, SM3: R-index performance) and the binary files for an R package: Cantor_Gero_ESM.pdf [file rsos140540supp1.docx]

**Supplementary Material to the article in the Royal Society Open Science:**

**The missing metric: Quantifying contributions of reviewers**

Maurício Cantor^1,3^ and Shane Gero^2,3*^

^1^Department of Biology, Dalhousie University, Halifax, Nova Scotia, B3H4J1, Canada

^2^Department of BioScience, Aarhus University, Aarhus, 8000, Denmark

^3^The authors contributed equally to this work

*Corresponding author: shane.gero@biology.au.dk

**SM1. Empirical and simulated data.**

Our modeling of the *R*-index was based on empirical and simulated data. The journals impact factor, *IF*, we obtained from the empirical exponential distribution (Journal Citation Reports, *JCR*, of the Science Citation Index, *SCI*). The editor’s score of excellence, *s*, we sampled from an empirical beta distribution (source journal: Marine Mammal Science), rescaled to be distributed between 0 and 1. The word count of manuscript, *w*, we generated by a normal distribution $N(6\cdot{10}^{3},2560\cdot{10}^{3})$, in which the large standard deviation ($\sigma=1600$) aimed to reflect a range of realistic manuscript lengths (about 200 to 12000 words) across disciplines and journal tiers. The number of revisions per referee, *n*, we drawn from a uniform distribution $U(1, 100)$ or set constant, depending on the model. Figure S1 illustrates a typical dataset used in the simulations. Finally, to test specific parameters of the index, we analyzed subsets of their distribution, for instance the first or last quantiles.


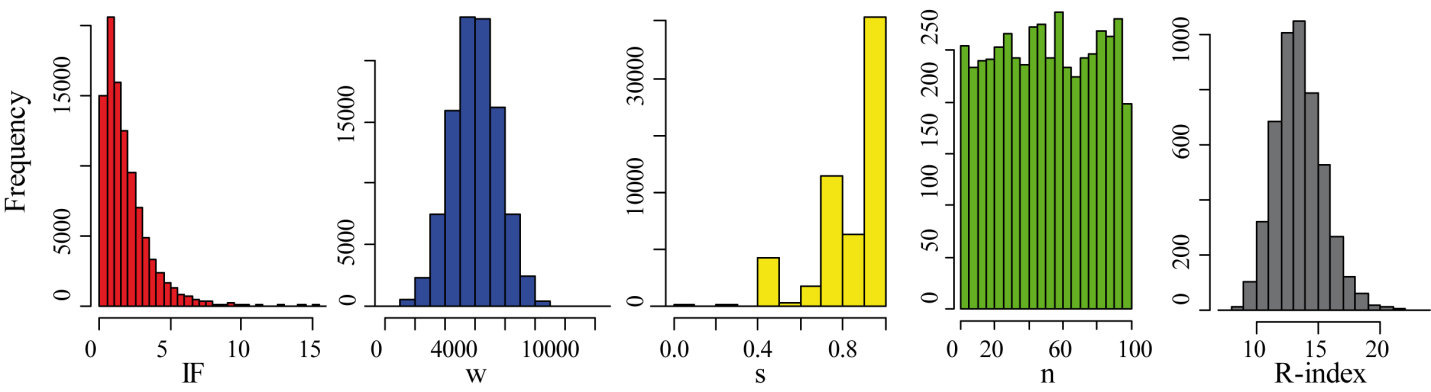


**Figure S1.** A typical simulated dataset. Number of reviewers *i* = 5000 and number of reviewed manuscripts *n* = 253000, *IF* following an exponential decay distribution as in the empirical data (one-sample Kolgomorov-Smirnov, *p*<0.01); *w* following a normal distribution; *s* following a beta distribution (*α* = 2.67, *β* = 0.45, *p* < 0.01); *n* following a uniform distribution. Under such conditions, the *R-*index is normally distributed across reviewers.

**SM2. Reviewers’ strategies and career stages**

To compare the *R-*index across stage careers and reviewer strategies (Figures 1 and 2 in main text), we delineated three career stages: early-career researchers (PhD candidates and post-docs), mid-career researchers (with more than 10 years after PhD and who likely have permanent positions) and lead researcher (i.e. professors, tenures, directors, deans). We assumed that the latter usually are invited to review for a broader range of journals, encompassing low-*IF* to top journals, and thus can be more selective. Consequently we further divided this category into two strategies: opportunist lead researchers, who review manuscripts only for top journals; and specialist lead researchers, who review only manuscripts on their area of expertise.

The reviews of early-career researchers were characterized by many (high *n*) moderately long manuscripts (mid-high *w*) submitted to low-rank journals (low *IF*) journals. Mid-career researchers reviewed several (moderate *n*) manuscripts of all lengths (normally distributed *w*) submitted to all types of journals (empirically distributed *IF*). Finally, opportunist lead researchers reviewed few (low *n*) and relatively short (moderate-low *w*) manuscripts for top journals (high *IF*), while specialist lead researchers reviewed few manuscripts (low *n*), but of all lengths (normally distributed *w*) and submitted to all types of journals (empirically distributed *IF*).

These two stereotypical strategies for lead researchers aimed to mimic the extremes of a broad range of possible reviewing habits. We further assessed if they did so, by simulating two mixed strategies, in which individuals could perform as both opportunistic and specialist by reviewing for either low- or high rank journals (empirically distributed *IF*), but differing in number of performed reviews and length of the manuscripts. Reviewers of the first mixed strategy reviewed few long manuscripts (low *n*, high *w*), while those of the second strategy reviewed many short manuscripts (high *n,* low *w*). The Figure S2 cross-validated the extremist strategies, as it shows that the *R-*index of mixed strategies indeed lay within the *R-*index of opportunist and specialist reviewers.


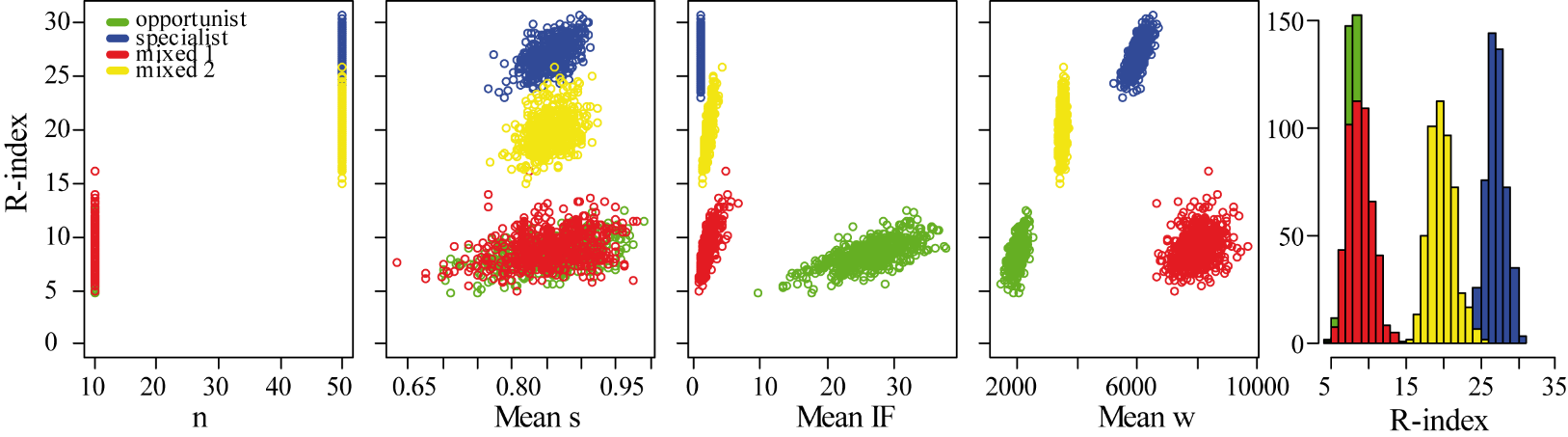


**Figure S2.** Characteristics of reviews by researchers with different strategies: opportunists, specialists and two mixed strategies. Scatter plots depict simulated *R*-index (y-axis) *vs.* mean index parameter (x-axis) for each reviewer *i* (colored circles). Histograms present the output *R*-index distributions showing that the main strategies considered in the text, opportunist and specialist, covered the extremes of review habits and mixed strategies are expected to fall in between them.

The sample size of reviewers in each of the four categories and the proportion of reviews they completed (*n*) were defined based on a recent online survey conducted by the Peerage of Science (http://www.peerageofscience.org/). This suggested that 54% of reviews are performed by early career researchers (42% postdocs and 12% PhDs); 32% by mid-career researchers; and 14% by lead researchers. However, the real-world population of researchers is likely to be composed by 38% of early-career researchers, 39% mid-careers and 23% leaders (http://www.peerageofscience.org/profs-vs-postdocs-in-peer-reviewing/). Therefore, our simulated population of 50,000 researchers and 2,875,000 manuscripts was composed by 27,000 early-career researchers reviewing 75 manuscripts each, 16,000 mid-careers reviewing 40 manuscripts each, 3,500 opportunist and 3,500 specialist lead-researchers reviewing 30 manuscripts each.

While the empirical data on review quality (*s*-score) suggest reviewers usually deliver reviews of good quality (beta distributed *s*-score; Figure S1), we further assessed the effect of quality of the review on the *R-*index outputs by setting different *s*-score profiles to the career stage categories with a stratified sampling of the empirical *s*-score distribution). Here, early-career researchers delivered high quality reviews (i.e. mainly high *s-* scores, drew from the 3^rd^ and 4^th^ quantiles of the empirical distribution), mid-career and specialist lead researchers delivering good reviews (mid *s*-scores, sampled from the 2^nd^ and 3^rd^ quantiles), and opportunist lead researchers delivering poor reviews (low *s*-scores, sampled from the 1^st^ and 2^nd^ quantiles of the empirical distribution). There are no empirical evidences that reviewers for top journals do a poor job; however, with the implementation of the *R-*index such strategy could arise as an attempt to game the system by providing many quick, poor reviews to boost one’s index. Our modelling aimed to evaluate how this strategy would perform, in order to assess how easily one could game the *R-*index.

**SM3. The *R-*index formulation.**

We formulated the *R-*index to reward reviewers proportionally to their contributions to the peer-review system, based on three parameters: the number of revisions (*n*), the length of the manuscript as a proxy for the time spent during the review (word count *w,* rescaled by 10^-4^ to make it less disparate across disciplines and journals), the impact factor of the journal the manuscript was submitted to as a proxy for the prestige and impact of the prospective paper, as well as the standing of the reviewer in the field (square rooted *IF* to be standardized across disciplines and career stages). In addition to quantity, we also want the *R-*index to promote quality of the revisions. Thus we introduced a score of excellence (*s*) to be given by the journal editors to each reviewed manuscript. The *s*-score ranges from 0 (very low quality) to 1 (exceptionally high quality). Some journals already have internal rankings of reviews, and we suggest here that ­*s-*score can and should be standardized across journals by averaging the scores of four qualities of the review, all which range between 0 and 1: a) punctuality: within or beyond the deadline set by the editor; b) utility to authors: are there constructive and specific comments for improving the work?; c) utility to editor: does the review address all facets of the manuscript—methodological and writing details, adherence to journal format—and is reported clearly and concisely?; and d) impact: to what degree did the review contribute to the decision made on the manuscript by the editor? Punctuality can be determined quantitatively by subtracting the proportion of days late from the amount of time given. The remaining three qualities can be scored on a 5-point scale (*e.g.* 0: unusable, 0.25: not useful, 0.5: adequate, 0.75: useful, 1.0: very useful). We propose editors use Multi-step Likert-type scales to quantify similar traits of the reviews themselves. Despite the potential subjectivity of the *s-*score, it is already the editors’ responsibility to evaluate the contribution a given review offered to the final paper and so we should trust their judgment. Therefore, the *s-*score is intended to promote high-quality reviews, rather than penalize poor-quality ones.

Clearly the larger the contribution to the peer-review system, with more and better reviews, the higher the reward should be. To check this, we compared the proposed *R*-index formulation with reduced, alternative versions of the index (Figure S3). The *R*-index always increases linearly with number of performed revisions, but when we included *IF*, this increment became disproportional: reviewing for high-*IF* journals would increase the *R*-index much faster (Figure S3). We constrained this effect by square-rooting *IF*, as well as by adding other parameters to reward time and effort invested in the review (*w, s*). Therefore, the slope of the curve decreased, making the *R*-index more egalitarian (blue line, Figure S3).


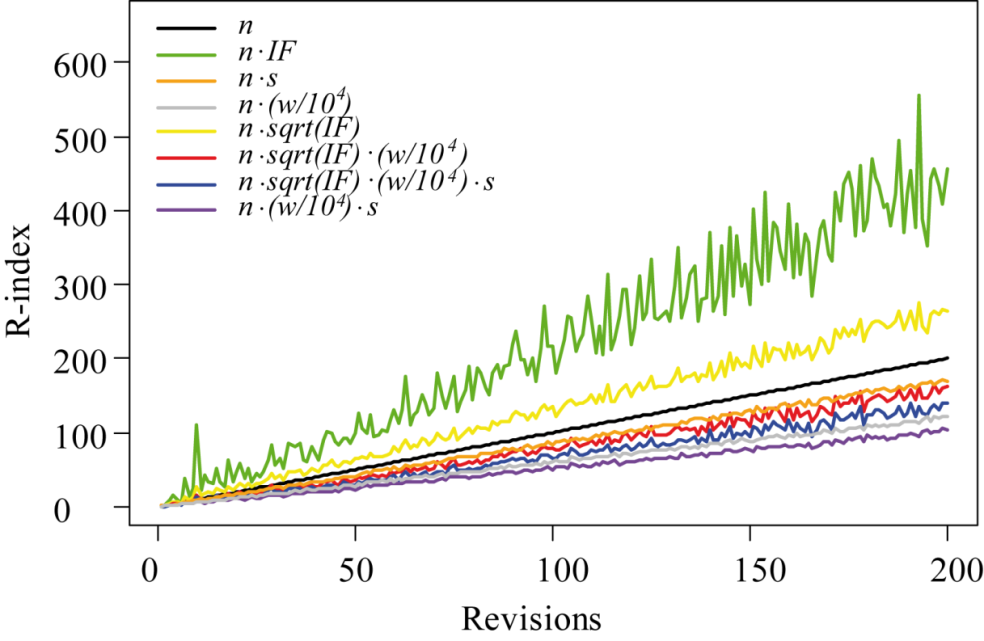


**Figure S3.** Different *R-*index formulations. The *R*-index increases linearly with number of performed revisions (*n*), with different slopes for different combinations of parameters in the formula (*IF*: impact factor; *w/10^4^*: rescaled word count; *s*: editor score). The proposed *R*-index increases gently with the number of revisions (blue line) by considering not only quantity (*n*), but the prospective impact of the manuscript (standardized by the square-rooted *IF* of the journal), the time spent during the review (given by the manuscript length) and quality of review report (ranked by the editor with the *s-*score).

Finally, we also assessed how each of the different *R-*index formulations performed with the reviewers’ strategies and career stages. The reduced alternatives changed the absolute scale of the index but amplified the disparities of reviewer of different career stages (Figure S4). Formulated in the proposed way, we believe the ­*R*-index captures many facets of the contributions through peer-reviewing, and so making the final outcome more balanced across career stages. Therefore, *R-*index has the potential of incentive participation of researchers of any stage in the peer-review system, with not only more, but also better, reviews of manuscripts.


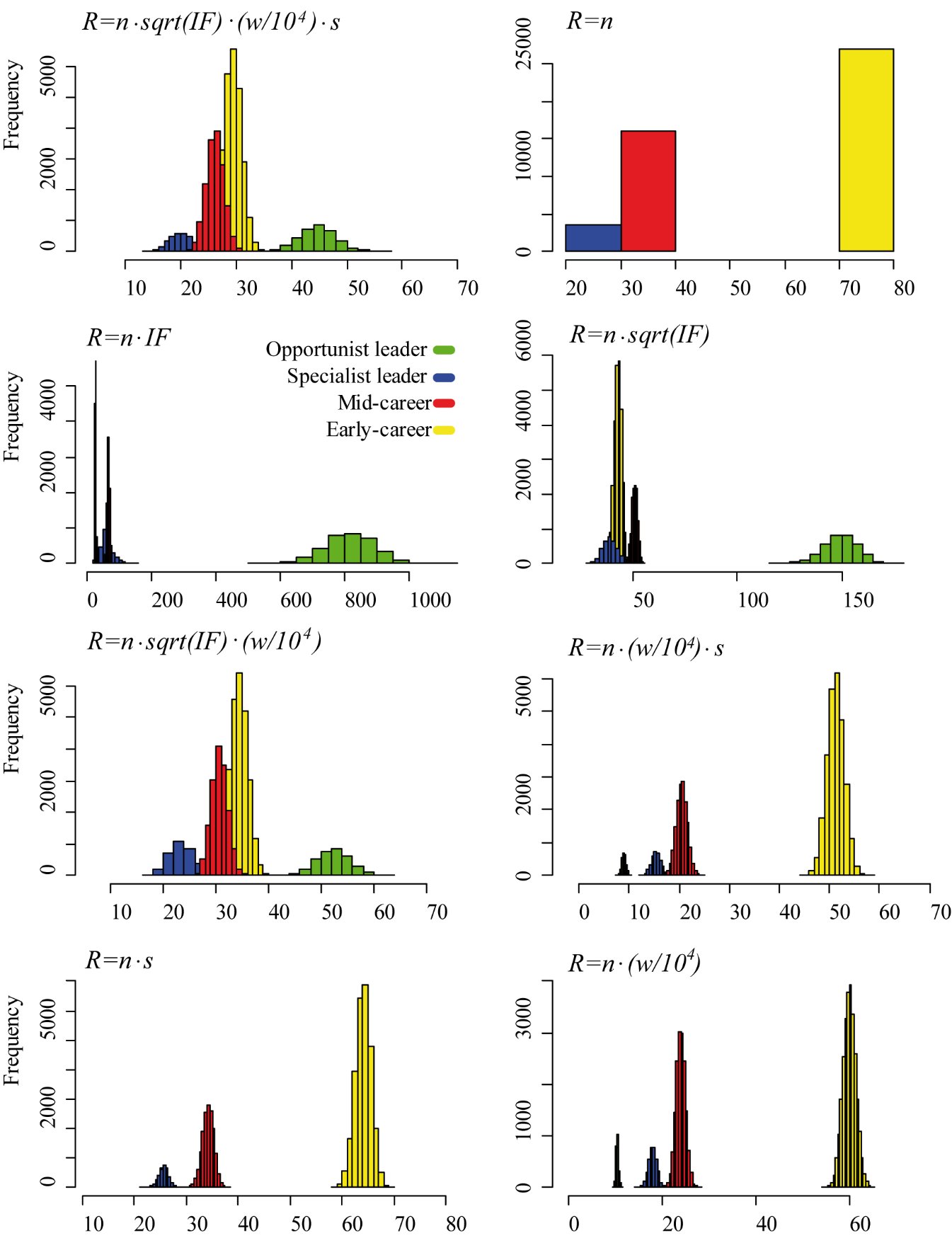


**Figure S4.** Performance of different *R-*index formulations in different simulated career stage profiles: opportunistic leader, specialist leader, mid-career and early-career researchers (see main text and ESM1 for details). The first histogram represent the outputs of the proposed *R-*index with four parameters (number of reviews, journal’s impact factor, manuscript length, and editor’s score – see main text for definition). The alternative, reduced indices are described in the Figure S3. The reduced alternatives change the absolute scale of the index and amplify disparities of reviewer of different career stages. On the other hand, the proposed *R-*index (first plot) captures many facets of the contributions through peer-reviewing, and so weights the number of reviewed papers making the final outcome more balanced. N.B.: where opportunistic leader output (green) is not shown it performed exactly as, and so is completely overlapped by, the specialist leaders (blue).

**SM4. *R-*index performance.**

To further explore *R*-index outputs, we simulated populations of 1000 reviewers with a fixed number of 50 reviews each, varying each parameter of the index independently (Figure S5). In all cases, the effect on the *R-*index was the similar: the more prestigious the journal (higher *IF,* Figure S5A), the longer the manuscript (higher *w,* Figure S5B) and the higher the quality of the review report (higher *s,* Figure S5C), the higher the reward. Therefore, in addition to the quantity of reviews one performers, the *R-*index accounts for the reputation of the manuscript in review and amount of time and effort invested in the review. When varying number of revisions *per capita*, the quality of the review is particularly decisive on the *R-*index output. Reviewers would need to complete 5 times as many poor reviews to achieve *R*-indices comparable to those submitting quality reviews (Figure S6).


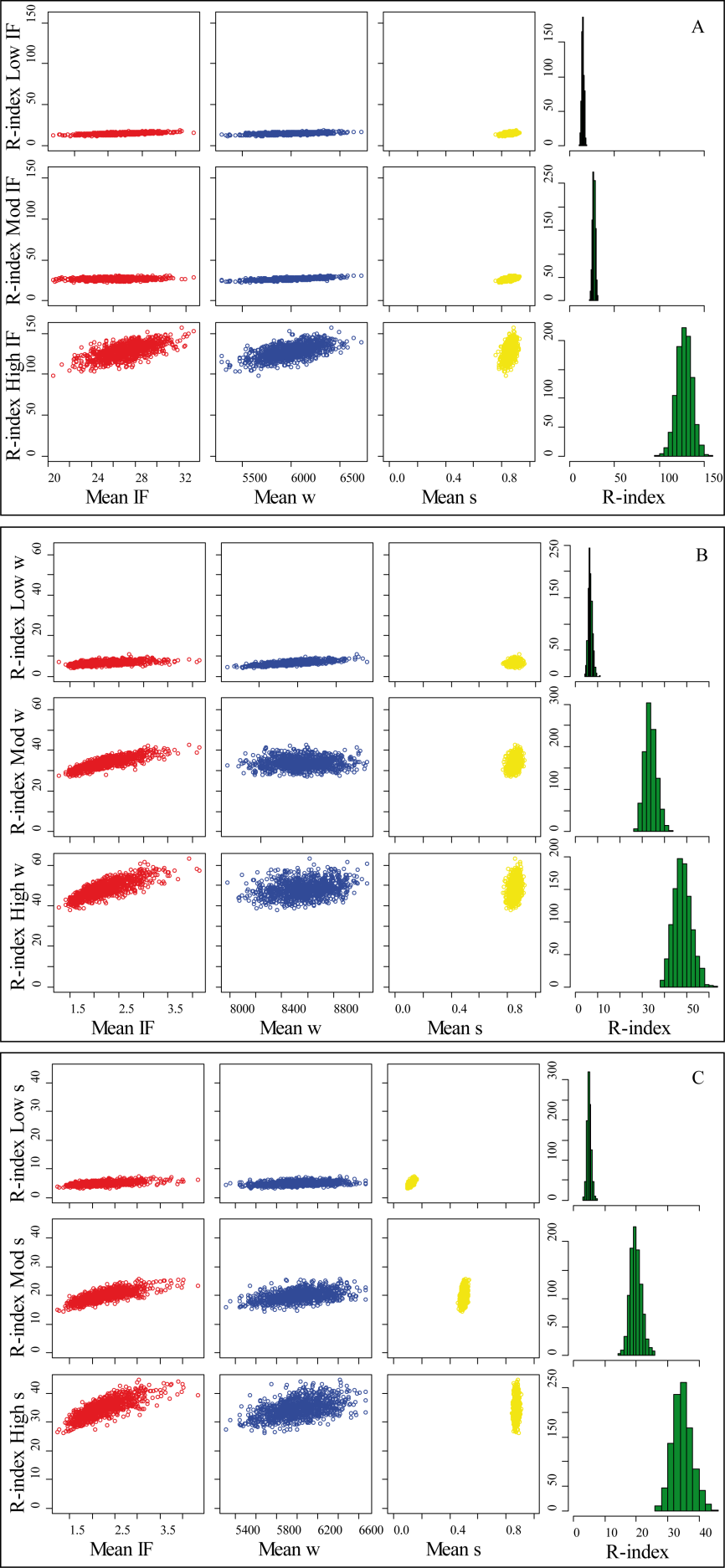


**Figure S5.** The effect of each parameter on the *R-*index. (A) journal’s impact factor IF to which the manuscript was submitted to, the manuscript length w and (C) the effect of editor’s feedback s. Scatter plots depict simulated *R-*index in the y-axis and mean parameter for each reviewer (colored circles) and histograms present the *R-*index distribution for the simulated scenario (each row of graphs). Datasets were simulated with the same number of reviews (*n* = 50) per researcher (*i* = 1000), but differed in: (A) the range of *IF* (first row represents a sample of low rank journals; second row mid-rank journals; third row top journals); (B) range of *w* (first row represents short manuscripts with range = 400-3000, mean = 1200, SD = 750 words; second row medium manuscripts, with range = 5500 - 6500, mean = 6000, SD = 200 words; third row long manuscripts with range = 7000 - 10000, mean = 8500, SD = 1200 words); and range of *s*-score (first row represents poor-quality reviews, range = 0 - 0.25; second row average quality, range 0.35 - 0.65; third row excellent reviews, range = 0.75 - 1). In all cases, the higher the target parameter, the higher the *R-*index: as *IF* (red scatter plots in A), *w* (blue plots in B) or *s* (yellow plots in C) increases, the output distributions of *R-*index (green histograms) shift to higher values.

**
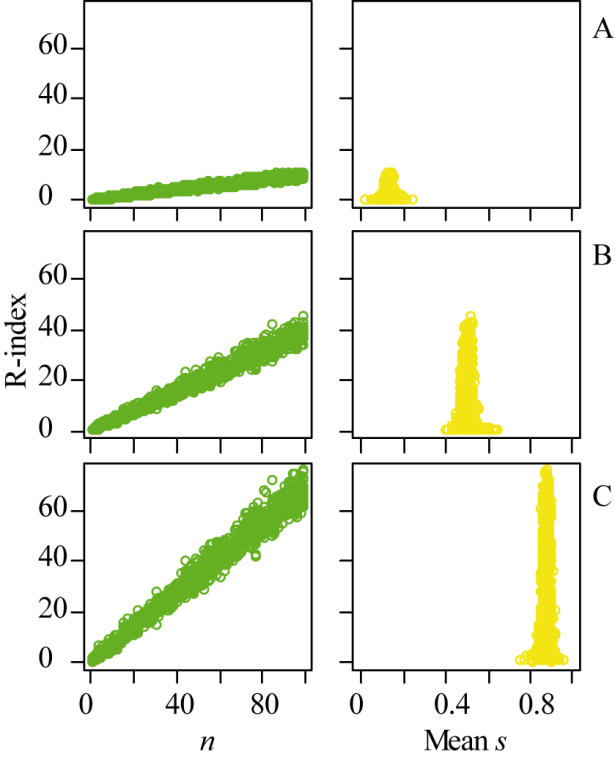
**

**Figure S6.** The importance of quality over quantity in the peer-review. *R-*index increases drastically with the quality of the review, given here by the mean score of excellence provided by the journals’ editors. We simulated three scenarios, in which 50733 manuscripts are reviewed by 5000 reviewers. Reviewers varied in quantity (number of reviews *n*) and quality (mean *s-*score) of reviews: (A) poor-quality reviews (*s* = 0 - 0.25), (B) average quality (*s* = 0.35 - 0.65), (C) excellent reviews (*s* = 0.75 - 1). In order to achieve the same *­R*-index = 10, one performing poor reviews would need to review 5 times more manuscripts (*n* = 100 in A) than on delivering high-quality reviews (*n* = 20 in C).

Finally, we explored how *R*-index would perform with the bloom of new online journals, which could be used to game the *R*-index. Assuming that new online journals have very low, if any, impact factor *IF*, we simulated 5,000 individuals reviewing 250,000 manuscripts from journals with impact factors drawn from the empirical distribution (SM1) and then compared the *R*-index outputs when the same amount of manuscripts with very low impact factor (*IF =* 0.001) is reviewed. In both cases, the *R*-index was proportionally driven by the number of reviews performed (*n*), while weighted by the manuscript length (*w*) quality of the review (*s*) (Figure S7). However, the range of the *R*-index was largely decreased for the mimicked online journals with very low *IF*s (note y-axis in Figure S7). Even as a debatable evaluation of a journal’s reputation, the *IF* would control for attempts to boost *R*-index.


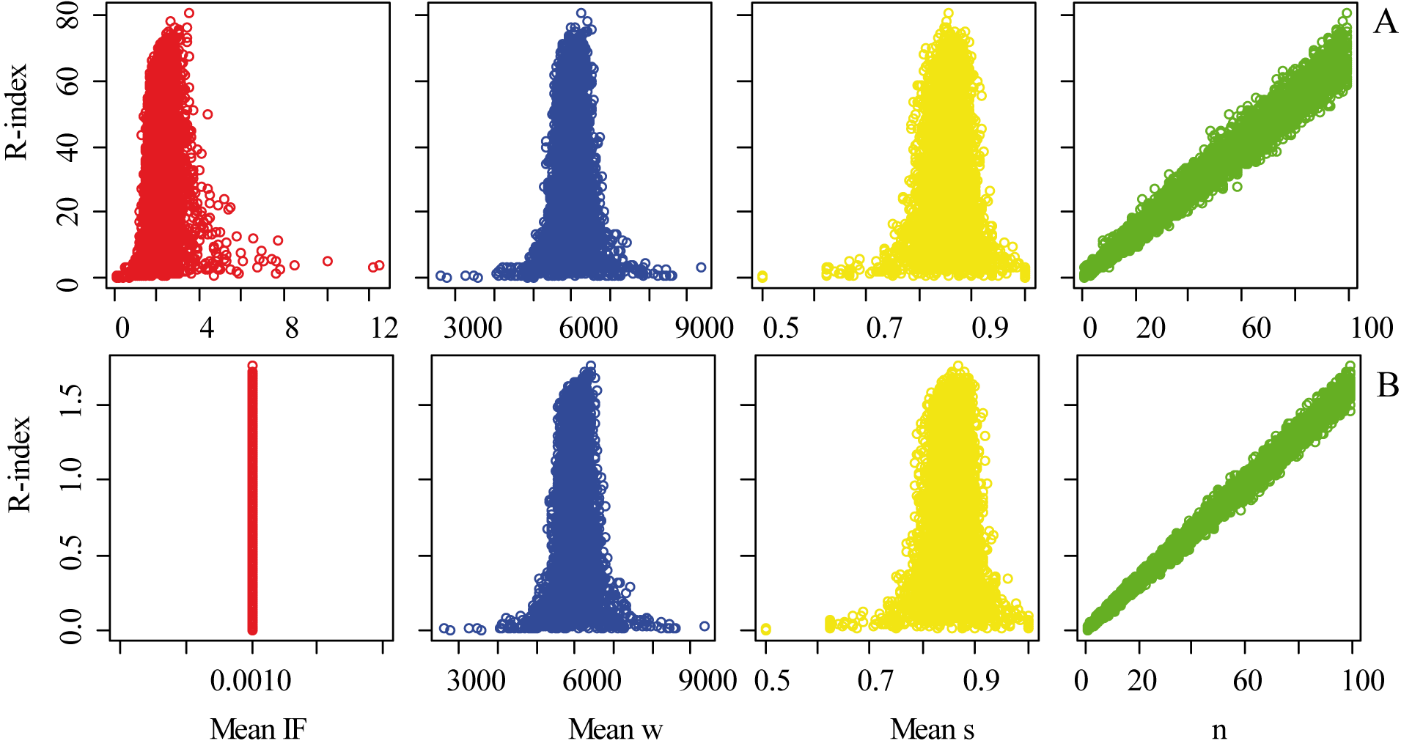


**Figure S7.** Performance of the *R-*index when (A) reviewing for journals of all types of impact factor (empirical distribution of *IF*) and (B) only reviewing for new online journals, here represented by manuscripts with very low impact factors (*IF* = 0.001). In both cases, the *R-*index was proportionally driven by the number of reviews performed (*n*), while weighted by the manuscript length (*w*) quality of the review (*s*), but in (B) the range of the *R-*index was largely reduced (note y-axis).
