# Supplementary material for: The missing metric: quantifying contributions of reviewers
Source: R Soc Open Sci. 2015 Feb 11;2(2):140540. doi: 10.1098/rsos.140540 (PMC4448813; doi:10.1098/rsos.140540)
Supplement: Supplementary methods Rindex_0.1.zip: R package Rindex [file rsos140540supp2.zip › Rindex/html/00Index.html]

R: Reviewing Reviewers' Rewards

# Reviewing Reviewers' Rewards

---

## Documentation for package ‘Rindex’ version 0.1

- DESCRIPTION file.

## Help Pages

|  |  |
| --- | --- |
| Rindex-package | Reviewing Reviewers' Rewards |
| bimoddist | Bimodal distribution |
| datagen | Data generator |
| minmax | Maximum and minimum R-index |
| plotsimu | Plotting simulation |
| rcindex | R-index for evaluating journal referees' contributions |
| rcindex.partial | Partial R-index for simulations |
| rcparplot | Plotting R-index vs parameter of different review strategies |
| rescale | Rescaling distribution |
| Rindex | Reviewing Reviewers' Rewards |
| varparplot | Plotting R-index outputs in four different scenarios |
| varparplot2 | Extracting R-index outputs in four different scenarios |
